# Unveiling the Proteomic Landscape of Extracellular Vesicles: Implications for Neurodegeneration and Neuroprotection

**DOI:** 10.1111/jnc.70350

**Published:** 2026-01-14

**Authors:** Berenice N. Bernal‐Vicente, Isaac Ponce, Emmanuel Ríos‐Castro, Perla Moreno‐Castilla, Luis B. Tovar‐y‐Romo

**Affiliations:** ^1^ Department of Molecular Neuropathology, Instituto de Fisiología Celular Universidad Nacional Autónoma de México Mexico City Mexico; ^2^ Genomics, Proteomics, and Metabolomics Unit National Laboratory of Experimental Services LaNSE, Cinvestav Mexico City Mexico; ^3^ Laboratory of Cognitive Resilience Centro de Investigación Sobre el Envejecimiento, Cinvestav Sede Sur Mexico City Mexico

**Keywords:** exosomes, extracellular vesicles, mass spectrometry, neurodegenerative diseases, proteomics

## Abstract

Extracellular vesicles (EVs) are instrumental mediators of intercellular communication and molecular exchange in neurodegenerative and neurovascular diseases. This review integrates recent advances in EV proteomics to elucidate their roles in Alzheimer's disease (AD), Parkinson's disease (PD), amyotrophic lateral sclerosis (ALS), traumatic brain injury (TBI), and ischemic stroke. Across these conditions, EVs carry disease‐relevant proteins that reflect and influence key pathological processes such as synaptic dysfunction, neuroinflammation, blood–brain barrier (BBB) disruption, and cell death. Proteomic profiling of brain‐ and biofluid‐derived EVs has uncovered specific biomarkers and signaling pathways, ranging from tau and α‐synuclein in AD and PD to mutant SOD1 in ALS and complement activation in stroke and TBI. Moreover, cell‐type‐specific EVs (e.g., from neurons, astrocytes, microglia, and stem cells) have been shown to exert either protective or deleterious effects, modulating apoptosis, axonal regeneration, and immune responses. Recent evidence highlights the translational potential of EVs as non‐invasive biomarkers and therapeutic vectors across multiple disorders. By mapping shared and divergent proteomic signatures in EVs, we review the mechanistic relevance and clinical utility of EVs in neurodegeneration and CNS injury.

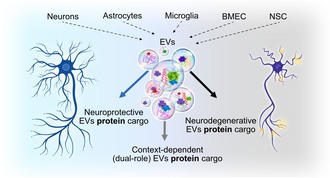

## Graphical abstract

Extracellular vesicles (EVs) are tiny, membrane‐covered packets that cells naturally release to send molecular information to one another. In the brain, they function as couriers that move proteins, lipids, and other signals between neurons, glial cells, and the blood–brain barrier. This review explains how studying the protein content of EVs helps scientists understand what is happening inside the brain during conditions such as Alzheimer's Parkinson's, amyotrophic lateral sclerosis (ALS), traumatic brain injury, and stroke. By analyzing EVs from blood, cerebrospinal fluid, or brain tissue, researchers can potentially detect early disease‐related changes, follow inflammatory processes, monitor the spread of harmful proteins, and identify molecules involved in either injury or repair. Because EVs travel through the bloodstream and can reach the brain, they are becoming valuable tools for earlier and more accurate diagnosis, and they are being considered as natural carriers for therapies that need to reach specific brain cells. EV proteomics is rapidly becoming a central strategy for both understanding and treating neurological disease.

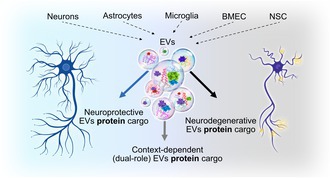

**Abbreviations**
AD: Alzheimer's disease
ALS: amyotrophic lateral sclerosis
BBB: blood–brain barrier
BMEC: brain microvascular endothelial cells
CSF: cerebrospinal fluid
CTE: chronic traumatic encephalopathy
ESI: electrospray ionization
EV: extracellular vesicles
hiPSC: human induced pluripotent stem cell
HPLC: high‐performance liquid chromatography
ISEV: International Society for Extracellular Vesicles
LC: liquid chromatography
LC–MS/MS: liquid chromatography–tandem mass spectrometry
LIT: linear ion traps
MALDI: matrix‐assisted laser desorption/ionization
MISEV: minimal information for studies of extracellular vesicles
MS: mass spectrometry
NFL: National Football League
NSCs: neural stem cells
PAGE: polyacrylamide gel electrophoresis
PD: Parkinson's disease
PFF: preformed fibrils
PRM: parallel reaction monitoring
QqQ: triple quadrupoles
QTOF: quadrupole–time‐of‐flight
RIPA: radio‐immunoprecipitation assay
SDS: sodium dodecyl sulfate
TBI: traumatic brain injury
TOF: time‐of‐flight
UC: ultracentrifugation

## 1 Introduction

Neurological disorders represent some of the most challenging and heterogeneous diseases in clinical medicine, often characterized by insidious onset, progressive degeneration, and limited therapeutic options. In this context, the search for early diagnostic biomarkers and novel mechanistic insights is urgent.

**FIGURE 1:**
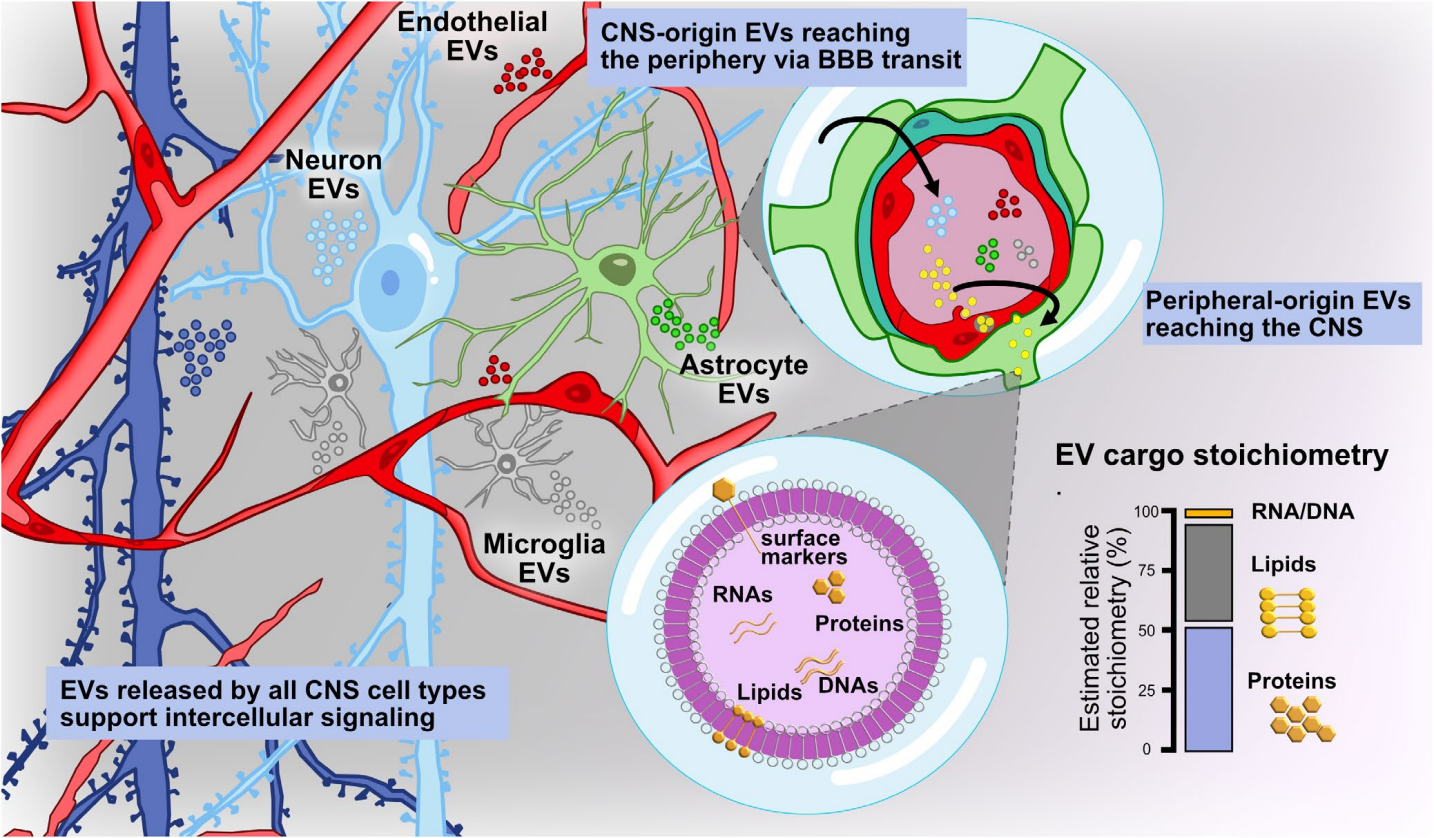
Central nervous system (CNS)‐derived extracellular vesicles (EVs) and their approximate cargo stoichiometry. EVs released by all major CNS cell types, including neurons, microglia, astrocytes, and brain microvascular endothelial cells (BMECs), contribute to local intercellular communication and can also cross the blood–brain barrier (BBB) bidirectionally, enabling CNS‐originated EVs to reach the periphery and peripheral‐origin EVs to access the CNS. The magnified vesicle schematic illustrates key cargo classes (proteins, lipids, RNAs, and DNA), and the accompanying bar depicts *a figurative relative cargo stoichiometry*, emphasizing the predominance of proteins and lipids (> 95% combined) and the trace‐level abundance of nucleic acids. Percentages represent conceptual approximations based on current literature rather than quantitative measurements (Chevillet et al. 2014; Skotland et al. 2019; Ghadami and Dellinger 2023).

Over the past decade, EVs have garnered substantial attention as mediators of intercellular communication in both health and disease. These small, membrane‐bound structures, enclosed by a lipid bilayer, are produced and released by all cell types into the extracellular space. EVs transport and deliver a wide array of biomolecules—including nucleic acids, proteins, lipids, carbohydrates, and metabolites—between cells (revised elsewhere Gurunathan et al. 2021). Their ability to be internalized by brain microvascular endothelial cells (BMECs), cross the BBB, and deliver molecular cargo to CNS cells (Dickens et al. 2017) has positioned them as central players in the pathophysiology of neurodegenerative and neuroinflammatory conditions (Figure 1).

Although historically viewed as uniform carriers of signaling cargo, EVs' molecular composition is now understood to be far more specialized and heterogeneous. Foundational work established their roles in intercellular communication, and their potential utility as disease biomarkers, while also highlighting major methodological obstacles in isolating, quantifying, and interpreting their in vivo functions (Zaborowski et al. 2015). Subsequent advances, particularly in lipidomics and proteomics, have reshaped this landscape by demonstrating that EV subtype identity, biogenesis pathways, and functional properties are tightly linked to specific molecular signatures. Detailed lipid‐focused studies have shown that exosomes and small EVs are enriched in cholesterol, sphingolipids, phosphatidylserine, ether lipids, and phosphoinositides, which collectively influence membrane curvature, fusion capacity, stability, and release dynamics (Skotland et al. 2017; Skotland et al. 2019). Lipid composition influences EV trafficking, tropism, and interactions with recipient cells. In some instances, like cancer cells, quantitative analyses have demonstrated that exosomes contain ~8.4‐fold more lipids per milligram of protein than the parent cells and approximately 65 lipid molecules per protein molecule, revealing a striking lipid‐dominated molecular ratio that suggests glycosphingolipids greatly outnumber exposed protein species on the vesicle surface (Llorente et al. 2013). This unusually high lipid‐to‐protein ratio may directly contribute to exosomal stability in extracellular environments and to their efficiency in long‐range communication. Consistent with these mechanistic insights, lipidomic profiling has been used to identify biomarkers in disorders such as AD, and engineering lipid composition is increasingly viewed as a promising means of enhancing EV targeting and therapeutic cargo delivery (Ghadami and Dellinger 2023).

Parallel proteomic dissection has refined EV classification beyond traditional exosomal markers. High‐resolution analyses demonstrated that tetraspanin‐enriched small EVs, considered bona fide exosomes, are marked specifically by syntenin‐1 and TSG101, whereas proteins historically assumed to be exosome‐restricted are actually distributed across large and medium EVs as well (Kowal et al. 2016). This molecular discrimination has been further sharpened by evidence that extracellular DNA and key RNA‐binding proteins such as Argonaute 1–4 are not components of small EVs; instead, extracellular DNA release operates through an amphisome‐dependent, exosome‐independent pathway, and annexin A1 serves as a reliable marker for microvesicles rather than exosomes (Jeppesen et al. 2019). Moreover, quantitative analyses of RNA cargo show that the majority of exosomes contain fewer than one copy of abundant microRNAs, challenging prevailing assumptions about exosome‐mediated miRNA transfer and indicating that only a minor subset of EVs contribute meaningfully to miRNA‐based communication (Chevillet et al. 2014). Furthermore, increasing the gene copy number in donor cells correlates with increased protein cargo in EVs, but not with corresponding RNA cargo. Thus, protein content rather than RNA content seems to respond to cellular changes in EV loading (Poudel et al. 2025).

Taken together, the current evidence strongly indicates that proteins, rather than RNA, constitute the predominant and functionally most relevant signaling cargo of EVs (Illustrated in Figure 1). Although early models emphasized miRNAs as key mediators of EV‐based communication, quantitative analyses show that most exosomes contain very low miRNA copies to exert meaningful biological effects, whereas proteins involved in membrane organization, trafficking, receptor engagement, metabolic regulation, and intracellular signaling are consistently abundant and mechanistically linked to EV function. The combined lipid–protein architecture of EVs further reinforces this view: lipids shape vesicle stability and targeting, but proteins ultimately execute the majority of downstream cellular responses. As the field continues to refine EV isolation and single‐vesicle analytics, it is becoming increasingly clear that protein cargo provides the most robust, reproducible, and biologically consequential signals delivered by EVs.

Proteomic analyses rely on different analytical techniques to investigate the structure and function of peptides and proteins. These include gel‐based methodologies such as sodium dodecyl sulfate‐polyacrylamide gel electrophoresis (SDS‐PAGE) in one or two dimensions, western blotting, and high‐performance liquid chromatography (HPLC). Additional approaches comprise protein microarrays, Edman degradation sequencing, enzymatic assays (e.g., ELISA), X‐ray crystallography, nuclear magnetic resonance spectroscopy, and mass spectrometry (MS) (Jiang et al. 2024).

Recent advances in MS–based proteomics have enabled the high‐throughput identification, quantification, and characterization of EV proteins with unprecedented sensitivity and specificity. Sensitivity enables detection of proteins across a wide dynamic range, while specificity is achieved through collision‐induced peptide fragmentation, generating unique fragment ions essential for peptide identification. This analytical power allows not only the detection and quantification (relative or absolute) of thousands of proteins, but also the characterization of post‐translational modifications and even the elucidation of protein–protein interaction sites. Currently, MS‐based proteomics is regarded as the gold standard for biomarker discovery across diverse biological samples (Thiele et al. 2024), including the molecular content—or “cargo”—in EVs. As such, this approach holds significant promise for early disease detection, improving the likelihood of effective therapeutic intervention. Nonetheless, the inherent complexity of various CNS disorders has precluded the identification of a single reliable diagnostic biomarker. To address this gap, research on EVs and their relationship to neurodegenerative diseases has intensified.

The proteomic profiling of EVs is facilitated by powerful analytical platforms such as liquid chromatography–tandem mass spectrometry (LC–MS/MS), often paired with isobaric labeling strategies (e.g., TMT, iTRAQ) or label‐free quantification. These tools permit the discovery of differentially expressed proteins and the mapping of post‐translational modifications that may modulate EV bioactivity and specificity. Crucially, proteomic data have started to delineate the cell‐type origin of EVs—neuronal, astrocytic, microglial—enhancing diagnostic precision and uncovering mechanistic disease pathways, such as the prion‐like propagation of misfolded proteins (e.g., tau, α‐synuclein, TDP‐43).

Despite these advances, the field faces considerable technical and conceptual challenges, including EV heterogeneity, lack of standardization in isolation protocols, and limited data reproducibility. Addressing these limitations is crucial for translating EV‐based proteomic signatures into clinically actionable biomarkers or therapeutic vectors. In this sense, adherence to guidelines from the International Society for Extracellular Vesicles (ISEV) and innovations in EV isolation and analysis—such as microfluidic technologies and single‐vesicle proteomics—are expected to enhance analytical precision and reproducibility.

In this review, we comprehensively examine the proteomic landscape of EVs in the context of neurological disease. We highlight methodological strategies for EV isolation and protein analysis, survey recent discoveries linking EV protein cargo to disease mechanisms and biomarkers, and discuss the translational potential of EVs for diagnosis, prognosis, and therapeutic delivery. By integrating biochemical, cellular, and systems‐level perspectives, we aim to provide a critical synthesis of this rapidly evolving field and outline future directions in EV‐based neurobiology. We focus on AD, PD, ALS, TBI, and stroke, representative neurological conditions in which EV biology has been most extensively characterized with respect to proteomic cargo, biomarker development, and mechanistic insights. These disorders collectively encompass neurodegenerative, neurovascular, and neurotraumatic etiologies, providing a broad yet coherent framework for evaluating how EVs reflect pathogenic processes across distinct forms of CNS injury. Although important EV research has also been conducted in additional conditions such as multiple sclerosis, epilepsy, or peripheral neuropathies, these fields exhibit substantial heterogeneity in methodological approaches, disease mechanisms, and available human‐derived EV proteomics datasets. To maintain a focused and integrative synthesis, we prioritized disorders with sufficiently robust and comparable evidence to support cross‐disease analysis. Nevertheless, many of the concepts discussed here are broadly applicable and continue to inform research in other neurological diseases.

## 2 Extracellular Vesicles

The term *exosome* was first introduced by Eberhard Trams in 1981 to describe molecules released from cells via budding processes (Trams et al. 1981). However, it was not until the late 1980s that Rose M. Johnstone identified nanoscale structures derived from reticulocytes released from the plasma membrane and termed *exosomes* (Johnstone et al. 1987). Following this discovery, scientific interest in EVs increased substantially, and numerous studies were initiated to explore their biogenesis and functional roles (Henne et al. 2011; Shen et al. 2011; Colombo et al. 2013). This surge in research also led to the recognition of the need for a standardized nomenclature (Gould and Raposo 2013).

EVs are primarily categorized based on their intracellular origin into two main types: exosomes and microvesicles, also referred to as microparticles or ectosomes. Exosomes, typically ranging from 30 to 150 nm in diameter, originate as intraluminal vesicles within multivesicular bodies, which subsequently fuse with the plasma membrane to release their contents into the extracellular space. In contrast, microvesicles, which range from 150 to 1000 nm, are formed through outward budding and plasma membrane fission before their release. To clarify and standardize this classification, ISEV has issued guidelines recommending that studies on EVs adopt precise terminology and consider specific characteristics when referring to exosomes or microvesicles (Welsh, Goberdhan, O'Driscoll, et al. 2024). According to these guidelines, vesicle size is a defining feature: *small extracellular vesicles* are generally < 100 nm, while *medium/large extracellular vesicles* exceed 200 nm in diameter. In 2019, a novel class of extracellular nanoparticles, termed *exomers*, was reported. These particles, measuring less than 50 nm in diameter, are known to contain nucleic acids and lipids that can be transferred to recipient cells. Nonetheless, exomers remain under investigation, and current knowledge about their biological roles is still limited (Zhang et al. 2019; Anand et al. 2021).

Although exosomes and microvesicles originate from distinct intracellular pathways, both can be secreted simultaneously and may share overlapping membrane‐trafficking routes (Russell et al. 2019). The release of EVs into the extracellular milieu has been documented across a wide range of brain cell types, including neurons, astrocytes, oligodendrocytes, microglia, BMECs, pericytes, and neural progenitor cells (Figure 1).

For a long time, these vesicles were primarily regarded as vehicles for cellular waste disposal, given their capacity to expel damaged DNA from cells (Takahashi et al. 2017). It is now well established that once secreted by their cell of origin, EVs can evade immune surveillance in the extracellular milieu and resist enzymatic degradation, particularly by RNases, thereby enabling them to reach proximal or distal target cells. In recipient cells, EVs can trigger intracellular signaling either via receptor–ligand interactions or through internalization mechanisms such as phagocytosis (Feng et al. 2010) or endocytosis, including clathrin‐mediated, caveolin‐dependent, or lipid raft–associated pathways (Gonda et al. 2019). Ultimately, EVs deliver their molecular cargo to recipient cells, modulating their physiological state, and thus function as potent mediators of intercellular communication (Ludwig and Giebel 2012; Colombo et al. 2014).

EVs are virtually present in all biological fluids, including blood, saliva, breast milk, cerebrospinal fluid (CSF), amniotic fluid, bile, urine, and semen. Exosomes derived from CNS cells, such as astrocytes, can be detected in distal organs, including the liver, spleen, and lungs (Dickens et al. 2017). Some researchers propose that this distribution is facilitated by transcytosis across the BBB, mediated by BMECs (Chen et al. 2016).

Due to their intrinsic characteristics—such as size, structure, molecular content or composition (including lipids, proteins, and carbohydrates), and even the identity of their cell of origin—EVs constitute heterogeneous populations (Witwer and Théry 2019). Currently, no definitive methodologies can clearly distinguish between exosomes and microvesicles, making it impossible to establish universal and specific markers for EV subtypes (Jeppesen et al. 2019; Welsh, Goberdhan, O'Driscoll, et al. 2024). Proteomic analyses of EVs have indicated that many membrane‐associated proteins are shared between exosomes and microvesicles, including CD9, CD81, CD63, and TSG101 (Kowal et al. 2016). Additional proteins implicated in EV identification include syntenin‐1, ADAM10, annexin XI, annexins (ANXA2, ANXA4), ALIX, integrins (ITGB1, ITGB4, ITGA6), flotillin, and EGFR (Jeppesen et al. 2019). More recently, it has been reported that both exosomes and exomers share common markers such as syntenin‐1, CD81, and hexokinase I (Zhang et al. 2019). Furthermore, exosomes are enriched in specific lipid raft–associated lipids, including cholesterol, ceramides, sphingolipids, and phosphoglycerides containing long saturated fatty acid chains (Choi et al. 2013).

Accumulating evidence indicates that sex influences the molecular cargo of EVs, shaping their lipid, protein, and RNA content in both physiological and pathological contexts. Comparative analyses of brain EVs reveal that repeated cocaine exposure alters the lipidome of male, but not female, mice, with increased GD1a gangliosides and reduced ceramide and LBPA levels, suggesting sex‐specific neuroprotective adaptations that may affect vulnerability to addiction and neurotoxicity (Landfield et al. 2021). Similar sex‐dependent patterns are observed in neurodegenerative conditions: in AD, the miRNA content of CSF‐derived EVs shows distinct signatures influenced by both sex and APOE‐e4 genotype, with females exhibiting higher levels of miR‐146b‐5p, miR‐150‐5p, and miR‐342‐3p, all linked to neuroinflammatory and autophagic pathways (Sandau et al. 2022). In vivo studies using hippocampal interstitial fluid EVs from APP/PS1 mice further demonstrate that microglial‐derived EV proteins respond to Aβ pathology in a sex‐specific manner—males displaying a more robust microglial proteome and fewer amyloid plaques, while females show enrichment of APOE‐ and CLU‐related proteins associated with AD progression (Pait et al. 2024). Beyond neurodegeneration, chronic SCI induces long‐term, sexually dimorphic EV responses: aged female mice display heightened microglial activation and ROS production, whereas males exhibit increased abundance of specific EV subtypes and markers, despite comparable behavioral impairments, suggesting divergent EV‐mediated neuroimmune mechanisms (Li et al. 2023). Broader analyses also highlight sex‐dependent variations in EV concentration, size, and composition across multiple systems, influenced by hormonal milieu, metabolic state, and inflammatory load, with implications for diseases ranging from cardiovascular disorders to neuroinflammation (Noren Hooten et al. 2022). Collectively, these findings substantiate that sex is a key biological variable determining EV cargo composition and signaling potential, shaping the functional landscape of intercellular communication in both health and disease.

## 3 Methods for Isolation and Identification of EVs

One commonly used method for identifying EVs is fluorescent labeling, which enables tracking EV trafficking and uptake in both live cells and fixed tissue samples. This technique relies on lipophilic dyes that incorporate into the EV membrane to facilitate visualization. Commonly used dyes include the PKH series and Dil, DiD, and DiR (Dehghani et al. 2020). Specifically, PKH26 and PKH67 have been employed to visualize EV internalization in pulmonary cells using scanning electron microscopy and laser scanning confocal microscopy (Reclusa et al. 2020). However, recent studies have reported that certain fluorescent lipophilic dyes may alter EVs' structure, function, and biodistribution (Simonsen 2019). Notably, PKH26 has been shown to increase EV size and modify their morphology as assessed by Nanosight nanoparticle tracking analysis (Dehghani et al. 2020). Therefore, fluorescent lipophilic molecules should be regarded solely as a preliminary approach for EV analysis and must be complemented by robust analytical methods for accurate isolation and quantification.

A major challenge in the study of EVs lies in developing effective isolation strategies due to their inherent heterogeneity, which includes small, medium, and large EVs and nanovesicles. It is therefore essential to rigorously assess the purity of EV preparations, as any contamination may interfere with their biological activity, particularly if intended for administration to biological samples (Paolini et al. 2016).

Differential ultracentrifugation (UC) remains the *gold standard* methodology for EV isolation (Théry et al. 2018) and is often combined with iodixanol or sucrose density gradient centrifugation (Greening et al. 2015; Lobb et al. 2015; Reclusa et al. 2020). This combined approach allows for the separation of EVs from common plasma contaminants such as lipoprotein particles and albumin or fibrinogen‐associated proteins (Onódi et al. 2018). It has been proposed that combining two or more isolation techniques can reduce the co‐purification of lipoproteins such as ApoB or ApoE, compared to using UC alone (Brennan et al. 2020). Additionally, coupling UC with density gradient centrifugation enables quantitative proteomic comparisons across isolated EV subpopulations (Kowal et al. 2016). Furthermore, two‐step centrifugation protocols yield EV suspensions with superior imaging quality compared to single‐step UC (Reclusa et al. 2020).

In addition to UC, several alternative techniques are employed for the isolation and purification of EVs, including ultrafiltration, immunoaffinity capture using specific surface markers, size‐exclusion chromatography (Abramowicz et al. 2018), gel filtration, and polymer‐based precipitation methods (Greening et al. 2015; Zeringer et al. 2015). Ultrafiltration has been shown to yield more efficient EV isolation from conditioned cell culture media compared to UC (Lobb et al. 2015). However, certain precipitation‐based methods—particularly those utilizing commercial kits—may contaminate residual matrix components. These can encapsulate exosomes, hindering their capacity to interact with or fuse to recipient cell membranes, ultimately compromising their functional potential (Paolini et al. 2016). All these procedures are summarized in Table 1.

**TABLE 1:** Summary of common EV isolation techniques, strengths, limitations, and suitability for neurological EV studies.

Technique	Key strengths	Key limitations	Notes/suitability for neurological EV biomarker work
Ultracentrifugation (UC)	Widely used; established protocols; capable of processing large volumes	Low recovery of some EV subsets; co‐pelleting of proteins/lipoproteins; potential vesicle damage from high g‐forces; time‐consuming, expensive equipment needed (Visan et al. 2022)	Good for initial discovery if volume allows, but care must be taken to assess purity and co‐contaminants
Size‐Exclusion Chromatography (SEC)	Gentle on vesicles; effective separation from soluble protein contaminants; reproducible, scalable; minimal specialized equipment (Sidhom et al. 2020)	May have lower yield compared to bulk concentration methods; column capacity limits volume; does not discriminate EV subtypes by biogenesis	Strong choice for downstream proteomics of human biofluids (e.g., plasma, CSF) in neurological settings
Affinity capture (Immuno‐affinity, chemical precipitation)	High specificity for defined surface markers; can enrich for subpopulations (e.g., CD63+, CD81+ EVs)	Marker bias limits representation of total EV pool; may miss vesicles lacking targeted markers; cost may be higher; potential for non‐EV binding	Useful when targeting a specific EV subtype (e.g., neuronal‐derived EVs) in biomarker studies, but requires validation of specificity
Pre‐concentration/Filtration (e.g., Tangential Flow Filtration (TFF))	Can process large volumes efficiently; minimal high‐g stress; faster throughput (Visan et al. 2022)	May concentrate non‐EV particles/proteins; downstream purification still required; filter clogging/fouling potential	Useful as a first step in large‐volume clinical sample processing (e.g., plasma) before SEC or other purification
Combined workflows (e.g., TFF + SEC, UC + SEC)	Leverages strengths of two methods (high yield + high purity); improves reproducibility and reduces contaminant load	More complex; higher cost and time; requires optimisation; method transparency crucial given possible workflow‐dependent biases	Recommended for high‐quality EV proteomics in neurological biofluids when sample volume and budget allow

Immunoisolation via flow cytometry enables the selective separation of exosomes by using antibody‐coated beads that target surface proteins, such as CD9, CD63, and CD81. It has been demonstrated that CD63‐positive small EVs represent a distinct subpopulation from those carrying CD9 or CD81 (Kowal et al. 2016). Furthermore, flow cytometry offers the advantage of sorting vesicles by size, enabling high‐precision isolation of EV subpopulations that express one or more markers characteristic of specific EV groups. With the advent of microfluidics, numerous platforms have been developed for EV isolation and characterization. These platforms are often based on previously established methods, such as immunolabeling or size‐based separation, but leverage microchannel designs that enable analysis of small sample volumes with greater precision than conventional approaches. Current microfluidic chips—based on acoustic, magnetic, electric, or immunological isolation—have become increasingly common and offer advantages in reducing processing time and cost (Contreras‐Naranjo et al. 2017; Wu et al. 2022).

A major obstacle in the EV field—particularly for clinical and translational applications in neurology—is the lack of harmonized workflows that allow reproducible and comparable results across laboratories. ISEV has repeatedly emphasized this challenge, and the most recent guidelines, “Minimal information for studies of extracellular vesicles (Welsh, Goberdhan, O'Driscoll, et al. 2024)” (Welsh, Goberdhan, O'Driscoll, et al. 2024), outline minimum experimental requirements for EV isolation, characterization, and reporting. These include the use of orthogonal validation strategies (e.g., particle quantification, protein markers from multiple EV categories, assessment of non‐EV contaminants), transparent reporting of pre‐analytical variables, and explicit documentation of isolation parameters that can profoundly influence EV yield and cargo. Despite these recommendations, methodological variability remains substantial across neurological EV studies, limiting cross‐study interpretation and biomarker reproducibility. There is a need to adopt standardized, ISEV‐aligned protocols and rigorous quality‐control measures—such as consistent particle‐to‐protein ratios, reproducible isolation workflows, and adherence to the MISEV framework—to enable meaningful comparisons between cohorts and accelerate the translation of EV‐based biomarkers and mechanistic insights into clinical practice.

## 4 Overview of EV Proteomics

We include this overview of MS methodologies as a practical guide for researchers in the EV‐neuroscience field, emphasizing critical technical aspects that ensure reliable and reproducible proteomic analyses. As previously mentioned, MS remains the cornerstone of proteomic analysis, enabling the comprehensive identification and quantification of proteins in complex biological samples, including EVs. A critical aspect of proteomic workflows—particularly in EV studies—is the choice of ionization method, which directly influences sensitivity, resolution, and compatibility with complex biological matrices.

The most commonly used ionization techniques for proteomic applications are matrix‐assisted laser desorption/ionization (MALDI) and electrospray ionization (ESI). In MALDI, the analyte is mixed with an excess of a UV‐absorbing matrix, typically a low‐molecular‐weight aromatic acid. Upon laser excitation, the matrix molecules sublimate and transition into the gas phase, absorbing sufficient energy to facilitate proton transfer to the analyte, resulting in predominantly singly charged ions (z = 1+). An electromagnetic field then accelerates these ions into a time‐of‐flight (TOF) analyzer, where they are separated according to their mass‐to‐charge ratio (m/z)—the key parameter used to infer protein identity. This ratio reflects the atomic mass of the ionized molecule in Daltons, with each distinct protein exhibiting a characteristic m/z signature (Thompson et al. 2003; Steen and Mann 2004).

Mass spectrometers typically consist of three primary components: an ionization source, a mass analyzer, and a detector. These instruments are often coupled to liquid chromatography (LC) systems, which separate peptides generated during enzymatic protein digestion, thereby increasing the number of ions analyzed and enhancing protein identification efficiency. However, MALDI is not compatible with online HPLC coupling, but HPLC fractions can be deposited onto a MALDI plate for subsequent analysis.

In contrast, ESI allows peptides to be desalted, concentrated, and ionized in‐line using nanoscale HPLC (nanoLC). The mobile phases employed in this technique are typically acidic, facilitating protonation of basic amino acids (e.g., lysine and arginine) within peptides. A 2–6 kV voltage, applied at atmospheric pressure, is used to induce aerosolization at the capillary tip. The resulting charged microdroplets are heated and exposed to an auxiliary gas, usually nitrogen, which reduces droplet size. Once a critical diameter is reached, Coulombic repulsion causes the droplets to explode, releasing gas‐phase ions (Ho et al. 2003). Importantly, ESI can generate multiply charged species, meaning that a single molecule may carry multiple charge states (e.g., z = 1+, 2+, or higher) (Steen and Mann 2004). This multicharging provides a significant analytical advantage: higher charge states confer greater kinetic energy, enhancing fragmentation efficiency (Glish and Burinsky 2008; Ríos‐Castro et al. 2020).

The development of gas‐phase biomolecule generation using MALDI and ESI has significantly accelerated the study of proteins through MS, enabling both “online” and “offline” LC–MS workflows that combine MALDI or ESI ionization sources with a wide range of mass analyzers. These include quadrupoles (Q), linear ion traps (LIT), TOF analyzers, Orbitraps, and hybrid configurations (i.e., systems integrating two or more analyzers), such as triple quadrupoles (QqQ), quadrupole–time‐of‐flight (QTOF), TOF/TOF, LIT–Orbitrap, and others. Hybrid mass spectrometers generally offer superior sensitivity and resolution, making them highly versatile for high‐throughput proteomic experiments (Glish and Burinsky 2008).

Among the most commonly used MS configurations in proteomics are MALDI‐TOF/TOF, LC‐MALDI‐TOF/TOF, LC‐ESI‐QTOF, and LC‐ESI‐Orbitrap (Glish and Burinsky 2008). Platforms incorporating Orbitrap analyzers are the most widely used and currently dominate the field (Steigerwald et al. 2024). Moreover, tandem LC–MS/MS systems are crucial for obtaining primary peptide sequence information, thereby facilitating protein identification. This technology permits high precision in qualitative and quantitative analyses (Abbas et al. 2018). Finally, parallel reaction monitoring (PRM) is a targeted mass spectrometry technique that isolates and fragments specific peptide precursors, enabling highly sensitive and accurate protein detection. It uses high‐resolution Orbitrap analysis to quantify fragment ions, offering superior specificity, sensitivity, and multiplexing capabilities compared to traditional methods like immunoblotting (Bezstarosti et al. 2024). Each analytical platform presents specific advantages and limitations that directly impact analytical robustness and, consequently, method reproducibility. These factors are crucial for detecting low‐abundance proteins and accurately quantifying them, particularly when identifying differentially expressed proteins, molecules with potential utility as biomarkers in clinical and translational medicine.

Multiple analytical techniques used in proteomic studies of EVs can be integrated with MS. Classic examples include PAGE‐based approaches, such as two‐dimensional gels, where protein “spots” are excised and enzymatically digested—a process known as in‐gel digestion—followed by MS analysis. Numerous qualitative and quantitative studies of EVs have relied on this methodology (Kitamura et al. 2018; Manek et al. 2018; Muraoka, Jedrychowski, et al. 2020). However, a significant limitation of this approach is its low digestion efficiency and limited peptide recovery (Vowinckel et al. 2014).

Alternatively, proteins dissolved in buffer can be enzymatically digested without prior separation via SDS‐PAGE gels. This technique, known as in‐solution digestion, eliminates gel‐based separation and allows the digestion of all proteins in a sample for analysis in a single injection. In‐solution digestion, typically performed using trypsin, is currently the most widely used method. It identifies proteins from many samples (Brewis and Brennan 2010). This approach is commonly coupled with LC–MS for peptide separation and detection. The choice of digestion strategy should be aligned with the study's goals and subsequent data analysis.

Several EV proteomics studies have adopted in‐solution digestion, which has revealed a broad spectrum of proteins present in EV samples (Tomlinson et al. 2015; Musunuri et al. 2016; Bonafede et al. 2019). This method's popularity is mainly due to its higher yield (Vowinckel et al. 2014) and its ability to facilitate the analysis of hydrophilic proteolytic peptides, which are often difficult to detect using conventional MS workflows (Betancourt et al. 2020). Moreover, in‐solution digestion has been shown to enhance peptide sequence coverage (≥ 99%), enabling the detection of highly hydrophilic regions—including short hydrophilic peptides (2–8 amino acids) and C‐terminal peptides—thereby revealing post‐translational modifications that would otherwise remain undetected using alternative methodologies (Espinosa et al. 2021).

One of the primary limitations in EV proteomics—particularly in MS analysis—is the presence of contaminants such as albumin, which can mask the detection of lower‐abundance proteins (Pellitteri‐Hahn et al. 2006). Additionally, high concentrations of salts, polyethylene glycol, detergents (e.g., SDS), cytotoxic chemical agents, and bovine or caprine sera (commonly used in mammalian cell culture) can significantly impair sample preparation for EV proteomic studies (Patel et al. 2019). One study employed MS‐based proteomics following UC isolation to compare five different protein extraction buffers and assess the EV protein cargo. Results showed that the RIPA and Tris–Triton X‐100 buffers yielded the highest numbers of proteins and peptides, with reproducibility rates of 86% for proteins and 72% for peptides in technical replicates (Subedi et al. 2019).

Advances in ionization methods, mass analyzer technologies, and proteomic workflows have significantly enhanced our ability to investigate the complex protein cargo of extracellular vesicles. The integration of optimized sample preparation protocols, such as in‐solution digestion, with high‐resolution MS platforms has enabled the sensitive and reproducible identification of EV‐associated proteins, even at low abundance. Nonetheless, technical challenges—including sample contamination, digestion efficiency, and ionization variability—remain critical considerations that can impact data quality and biological interpretation.

## 5 EV Proteomics in Neurological Conditions

The evolution of proteomic technologies has considerably advanced the study of EV cargo, enabling not only comprehensive compositional mapping but also the identification of disease biomarkers. In translational research, EV proteomics has opened new opportunities for investigating CNS pathologies through peripheral biofluids, offering a minimally invasive alternative for accessing brain‐derived molecular signatures when direct sampling of neural tissue is not feasible. This has been particularly impactful in neurological diseases, where EVs serve as both mechanistic mediators and potential diagnostic tools. In Table 2, we present a summary of proteomic studies conducted on samples from patients with different neurological conditions. Importantly, the modulatory effects of EVs, including attenuation of neuroinflammation and preservation of synaptic integrity, align with established definitions of neuroprotection, whereby pathological cascades are altered to safeguard neuronal structure and function under disease stress. Supporting this, proteomic profiling of astrocyte‐derived EVs reveals enrichment of neuroprotective proteins, including HSP70, HSP90, Annexin V, Complement C3, Nucleophosmin, and glycogen phosphorylase, which orchestrate stress responses, metabolic adaptation, and survival pathways relevant to neuroprotection (Bernal Vicente et al. 2025).

**TABLE 2:** Proteomic studies conducted on samples from patients with neurological conditions.

Related disease	Sample type	Analytical approach	Mass spectrometer	Methodology	Acquisition method	Identification algorithm	Proteins detected	References
AD	Protein from temporal neocortex samples	Relative quantification “label‐free” by peak intensity	Orbitrap LTQ (Thermo Scientific)	LC–MS/MS	DDA	X! Tandem	Two sets, 689 and 724	(Musunuri et al. 2016)
AD	Exosomes from neurons expressing a mutant Tau derived from pluripotent stem cell (iPSC)	Relative quantification “label‐free” by peak intensity	Orbitrap Q‐Exactive (Thermo Scientific)	LC–MS/MS	DDA	PEAKS	592	(Podvin et al. 2020)
AD	EVs from human cerebrospinal fluid	Relative quantification “label‐free” based in top3 intensity	Synapt G2‐S (Waters Corp.)	Liquid chromatography coupled with Ultra Definition Multiplexed MS/MS (LC‐UDMS^E^)	DIA	Algorithm performed by (Li et al. 2009) content in Protein Lynxs Global Server (PLGS) software	Two cohorts, 613 total proteins	(Chatterjee et al. 2023)
AD	EVs from human plasma	Relative quantification using 10 plex Tandem Mass Tag (TMT)	Orbitrap Fusion Lumus (Thermo Scientific)	LC–MS/MS	DDA	SEQUEST content in the Proteome Discoverer Software *v*2.2	672	(Zhang et al. 2024)
ALS	EVs from CSF	Relative quantification “label‐free” by	Orbitrap Elite ion trap (Thermo Scientific)	LC–MS/MS	DDA	Algorithm performed by (Li et al. 2009) content in Progenesis QI for proteomic software	334	(Hayashi et al. 2020)
ALS	EVs from CSF	Relative quantification “label‐free”	Orbitrap Fusion Lumus (Thermo Scientific)	LC–MS/MS	DDA	Mascot v2.5.1	1020	(Thompson et al. 2020)
ALS	EVs from human plasma	Relative quantification “label‐free” by peak intensities	Q‐Exactive (Thermo Scientific)	LC–MS/MS	DDA	Andromeda content in MaxQuant software *v*1.6.2.3	107	(Pasetto et al. 2021)
CTE	EVs from CSF	Relative quantification using TMT	LTQ‐Orbitrap Fusion Lumos (Thermo Fisher Scientific)	LC–MS/MS	DDA	SEQUEST	429	(Muraoka et al. 2019)
Dementia with Lewy Bodies	EVs from platelet‐free plasma	Relative quantification “label‐free” by iBAQ	Orbitrap LTQ‐XL (Thermo Scientific)	LC–MS/MS	DDA	Mascot and Andromeda	Two sets: 240 and 540.	(Gámez‐Valero et al. 2019)
HIV‐associated neurocognitive disorders (HAND)	EVs from CSF	Relative quantification “label‐free” by peak intensity	LTQ Orbitrap Velos Pro ion‐trap (Thermo Scientific)	LC–MS/MS	DDA	SEQUEST	Two analyses, 2727 and 1626	(Guha et al. 2019)
PD	EVs from serum	Relative quantification “label‐free” by peak intensity	LTQ Orbitrap Velos (Thermo Scientific)	LC–MS/MS	DDA	Algorithm performed by (Li et al. 2009) content in Progenesis QI for proteomic software	1033	(Tomlinson et al. 2015)
PD	EVs from plasma	Bottom‐Up proteomics	MALDI‐TOF/TOF 4800 plus (AB Sciex)	2DE‐DIGE in combination with Tandem MS/MS	DDA	Paragon (Protein Pilot)	8	(Kitamura et al. 2018)
PD	Serum exosomes	Relative quantification “label‐free” by peak intensity	Orbitrap Q‐Exactive (Thermo Scientific)	LC–MS/MS	DDA	Andromeda	429	(Jiang et al. 2019)
PD	Urinary extracellular vesicles	Relative quantification “label‐free” by spectral counting	Orbitrap LTQ‐XL and Orbitrap Velos (Thermo Scientific)	LC–MS/MS	DDA	SEQUEST	1000	(Wang et al. 2019)
PD	EVs from plasma	Protein identification by Western Blot	NA	Antibody‐based methods	NA	NA	3 (SNAP‐25, GAP‐43, Synaptotagmin‐1)	(Hong et al. 2024)
PD	EVs from plasma	Relative quantification using 10 plex Tandem Mass Tag (TMT)	Orbitrap Fusion Lumus (Thermo Scientific)	LC–MS/MS	DDA	SEQUEST content in the Proteome Discoverer Software	555	(Zhao et al. 2025)
Stroke	EVs from serum	Relative quantification “label‐free”	Q‐Exactive HFX (Thermo Scientific)	LC–MS/MS	DDA	MASCOT search algorithm v2.6 content in Genedata Refiner MS Software *v*13.0.1	1288	(Qadri et al. 2024)
Stroke	EVs from plasma	Relative quantification “label‐free”	Orbitrap Fusion Lumus (Thermo Scientific)	LC–MS/MS	DIA	Spectronaut v18 or 19	556	(Reymond et al. 2025)
TBI	EVs from CSF	Bottom‐Up proteomics	Orbitrap LTQ‐XL (Thermo Scientific)	SDS‐PAGE in combination with LC–MS/MS	DDA	SEQUEST, X! Tandem	92 controls, 466 in TBI	(Manek et al. 2018)
TBI	EVs from plasma	MACSPlex analysis	NA	Antibody‐based methods and flow cytometry	NA	NA	Two panels, Panel A 39 proteins, panel B 21 proteins	(Schindler et al. 2024)

### 5.1 Parkinson's Disease (PD)

In PD, where the central pathology involves α‐synuclein aggregation and dopaminergic neurodegeneration, EVs serve as surrogates of ongoing neuropathological processes. A growing body of evidence, including systematic reviews and meta‐analyses, has demonstrated that α‐synuclein, particularly its oligomeric and phosphorylated species, is significantly enriched in neuronal EVs isolated from the plasma of PD patients (Xylaki et al. 2023). The use of immunoprecipitation techniques targeting neuronal markers, such as L1CAM, enhances the specificity of EV isolation, allowing differentiation of brain‐derived cargo from peripherally derived cargo. Moreover, a recent study has demonstrated a compelling link between α‐synuclein aggregation in the CSF of PD patients and LRRK2 mutations, highlighting the role of genetic risk factors in disease pathogenesis (Cao et al. 2023). In this context, ongoing efforts are directed toward both diagnostic and therapeutic innovation, including the exploration of LRRK2 content in EVs as a promising biomarker for PD (Vissers et al. 2023).

Mechanistic models using preformed fibrils (PFFs) have provided insights into the pathological propagation of α‐synuclein, recapitulating the Lewy body pathology characteristic of PD (Gómez‐Benito et al. 2020). These aggregates are known to disrupt mitochondrial membranes and trigger neurotoxicity, particularly in the substantia nigra pars compacta. It has been demonstrated that microglia exposed to PFFs release exosomes enriched in α‐synuclein, which facilitates the transfer of pathogenic α‐synuclein to neurons, promoting retrograde axonal degeneration along the nigrostriatal pathway (Guo et al. 2020).

Further supporting the diagnostic potential of EVs, a study employed 2D‐DIGE and MALDI‐TOF/TOF to identify differential protein expression, including clusterin, complement subcomponent C1r, and apolipoprotein A1, in exosomes from PD patients at Hoehn and Yahr stages II and III (Kitamura et al. 2018). These proteins were distinctly regulated compared with exosomes from healthy controls, and notably, apolipoprotein A1 became a candidate biomarker, in agreement with earlier ELISA‐based studies (Qiang et al. 2013). Such findings stress the potential of EV‐associated proteins for monitoring PD progression. However, it is important to note that this approach was limited to the most abundant proteins resolved on 2D‐SDS gels, potentially overlooking low‐abundance proteins of biological significance.

Complementing these approaches, Wang and colleagues reported that proteins such as SNAP23 and calbindin were differentially present in urinary EVs from a cohort of 28 PD patients, enabling disease prediction with 86% accuracy (Wang et al. 2019). Moreover, by analyzing 138 urine samples from various cohorts, including healthy individuals, non‐manifesting carriers of the LRRK2‐G2019S mutation, idiopathic PD patients, and LRRK2 PD patients, a study identified and quantified 4476 unique proteins and 2680 unique phosphoproteins (Hadisurya et al. 2023). Several proteins and phosphoproteins were elevated in patients with PD, particularly those involved in pathways such as autophagy, neuronal cell death, and neuroinflammation. Using machine learning, six top biomarkers were identified, yielding high predictive accuracy (AUC of 94.3%) for PD diagnosis. Validation experiments using PRM‐MS and Western blot confirmed the upregulation of key biomarkers, such as HNRNPA1 and PCSK1N. These findings highlight the potential of urinary EVs as a non‐invasive source for PD biomarkers, offering a promising avenue for early diagnosis and intervention.

Finally, a recent study provides a comprehensive proteomic comparison between non‐purified CSF and CSF‐derived EVs from patients with AD, PD, PD dementia, dementia with Lewy bodies, and PD with mild cognitive impairment (Hirschberg et al. 2023). Using label‐free MS, the researchers identified a greater number of differentially expressed proteins in CSF‐derived EVs (276) compared to non‐purified CSF (169), with minimal overlap between datasets. This suggests that CSF‐derived EVs may be more suitable for biomarker discovery due to their narrower dynamic range and reduced abundance of common proteins. The same study identified 39 promising biomarkers in non‐purified CSF and 37 in CSF‐derived EVs, which could aid in differential diagnosis and therapeutic development for neurodegenerative diseases. Notably, CSF‐derived EVs showed potential for identifying markers of neurodegeneration, while non‐purified CSF offered practical advantages for targeted proteomics. These findings highlight the complementary nature of both sample types and emphasize the need for further validation in larger cohorts, as well as the exploration of less‐invasive biofluids, such as plasma. The comparison between non‐purified CSF and CSF‐derived EVs raises an important question: which fraction better captures disease‐relevant information? While CSF‐derived EVs offer the advantage of a reduced dynamic range and enrichment of less abundant, potentially disease‐specific proteins, non‐purified CSF contains many high‐abundance proteins that may also reflect ongoing pathological changes. Indeed, some candidate biomarkers appear more abundant in non‐purified CSF, suggesting that bulk CSF measurements may, in some cases, provide a closer approximation of the overall disease state. Conversely, EVs likely highlight a distinct subset of proteins that are actively secreted or packaged by specific cell types, thereby offering mechanistic insights into disease propagation and progression. These complementary features suggest that EV‐based and non‐purified CSF proteomics should not be viewed as mutually exclusive but rather as parallel approaches: non‐purified CSF may serve as a more sensitive tool for monitoring global disease burden, whereas EV‐derived cargo could provide cell‐specific, mechanistic biomarkers with higher diagnostic precision. Future studies integrating both fractions, ideally in longitudinal cohorts, will be essential to determine how each contributes to tracking disease onset and progression in PD.

### 5.2 Alzheimer's Disease (AD)

AD is the most common form of dementia and is pathologically characterized by Aβ plaques, neurofibrillary tangles composed of hyperphosphorylated tau, progressive synaptic dysfunction, and neuronal loss. These hallmarks are accompanied by neuroinflammation, mitochondrial impairment, and lipid metabolism disturbances, which together drive cognitive decline and neurodegeneration. The study of EVs in AD offers opportunities to identify biomarkers that capture these diverse processes and to clarify mechanisms underlying disease progression. EVs from AD brain tissue, CSF, and plasma show distinctive molecular signatures compared to controls. Elevated levels of pathological proteins such as phosphorylated tau (pS396, pT181, pT231) and amyloid‐beta (Aβ1–42) have been consistently identified in AD‐derived EVs (Muraoka, Jedrychowski, et al. 2020). In addition, alterations in proteins related to gliosis, mitochondrial dysfunction, cholesterol accumulation, oxidative stress, and synaptic loss further support their involvement in neurodegeneration.

Proteomic analyses have revealed that glial‐specific proteins are enriched in AD EVs, whereas control samples show higher levels of neuron‐specific proteins, indicating astrocytic and microglial activation in AD. In fact, astrocyte‐derived EVs show the strongest association with AD pathology, with markers such as integrin‐β1 (ITGB1) and proteins from the M7 reactive astrocyte module tightly linked to amyloid and tau pathology. Similarly, microglial EVs exhibit a loss of homeostatic markers (e.g., P2RY12, TMEM119) and an upregulation of disease‐associated markers (e.g., TREM2, FTH1), tau, and GTPases, along with lipidomic changes including increased free cholesterol and reduced DHA‐containing species, indicating endolysosomal and membrane remodeling defects. These molecular alterations suggest that EVs from different CNS cell types may differentially contribute to AD progression (Cohn et al. 2021; You et al. 2022).

Moreover, DEPs have been identified in EVs from AD patients. For example, ANXA5, VGF, GPM6A, and ACTZ were shown to differentiate AD‐derived brain EVs from controls with 88% accuracy via machine learning, and ANXA5 levels correlated with Braak stage, a widely used neuropathological classification that stratifies tau pathology from early transentorhinal involvement (I–II), through limbic stages (III–IV), to widespread neocortical pathology (V–VI), underscoring its diagnostic potential (Muraoka, DeLeo, et al. 2020). Other identified candidates include ORM2, RBP4, HYDIN, and S100A8 in plasma EVs, the latter shown to regulate Aβ aggregation via EVs and demonstrating diagnostic value (AUC = 0.744). S100A8 downregulation in AD patient EVs was reversed in EVs from Aβ‐treated neuronal cells, and siRNA knockdown experiments reduced Aβ aggregation, particularly in EV fractions (Zhang et al. 2024; Nielsen et al. 2021). In this regard, Aβ aggregation in AD is influenced by multiple mechanisms, including: (i) the cross‐linking of coagulation factor XIII and Aβ into stable multimers resistant to proteolytic breakdown, contributing to aggregation and deposition along the cerebral vasculature in cerebral amyloid angiopathy, (ii) BBB dysfunction allowing Aβ to leak into circulation, exacerbating aggregation, and (iii) platelets releasing Aβ stored in α‐granules, while peripheral immune cells infiltrating the brain may adopt an inflammatory phenotype, promoting neuroinflammation and Aβ accumulation (Nielsen et al. 2021). Proteins like transthyretin (TTR) and retinol‐binding protein 4 (RBP4) transport Aβ and retinols, potentially reducing neurotoxicity and inhibiting oligomerization and chronic neuroinflammation, driven by activated microglia and astrocytes, further impair Aβ clearance and promote aggregation (Nielsen et al. 2021). These interconnected processes highlight the multifactorial nature of Aβ aggregation in AD.

In CSF‐derived EVs, multiple proteomic studies have revealed significant molecular shifts across AD stages. More than 2500 proteins have been cataloged, and several—such as HSPA1A, NPEPPS, and PTGFRN—show progressive dysregulation from mild cognitive impairment (MCI) to AD. PTGFRN, in particular, correlates with amyloid plaque and neurofibrillary tangle scores (Muraoka, Jedrychowski, et al. 2020). Complement component C1q was significantly increased in early MCI due to AD and validated by ELISA, reinforcing the role of neuroinflammation and synaptic loss in early pathology (Chatterjee et al. 2024). Similarly, cathepsin B (CatB) emerged as a biomarker, with its levels in CSF and plasma EVs showing inverse correlation with CSF Aβ42 and aligning with amyloid load, suggesting its role in Aβ metabolism (Yuyama et al. 2024).

Novel methods such as ExoSORT now enable immunoaffinity capture of neuron‐derived EVs (NDEVs) from plasma using GAP43 and NLGN3. These NDEVs have been shown to carry elevated levels of pT181‐tau and Aβ42 while exhibiting depletion of synaptic proteins including proBDNF, GluR2, PSD95, and Syntaxin‐1. A composite biomarker panel based on these cargo changes achieved 94.7% sensitivity in distinguishing AD patients, demonstrating both diagnostic robustness and scalability (Eitan et al. 2023).

EVs also appear to propagate pathology. Brain‐derived EVs from AD and frontotemporal dementia patients and mouse models induced memory impairments and behavioral changes in both wild‐type and tau‐transgenic animals. These vesicles carry pathological tau and Aβ proteins and are enriched in molecules linked to synaptic dysregulation. Tau is notably localized to the EV lumen, suggesting a vesicle‐mediated mechanism for the intercellular spread of pathology (Bodart‐Santos et al. 2023).

More broadly, EV proteomics has identified key pathological pathways—including amyloid metabolism, tau phosphorylation, synaptic dysfunction, oxidative stress, and neuroinflammation. New platforms such as microfluidics‐based isolation, single‐vesicle analysis, and multi‐omics integration offer refined resolution for biomarker discovery and disease stratification. While EVs hold great promise for early diagnosis, monitoring, and therapeutic delivery in AD, challenges in standardization, validation, and regulatory approval remain significant hurdles to clinical translation (Pei et al. 2024).

### 5.3 Amyotrophic Lateral Sclerosis (ALS)

ALS is a progressive neurodegenerative disease marked by the selective degeneration of upper and lower motor neurons in the cerebral cortex, brainstem, and spinal cord, ultimately leading to irreversible paralysis (Tovar‐Y‐Romo et al. 2009). Although its etiology is not fully understood, evidence suggests that astrocytes expressing the ALS‐linked G93A‐SOD1 mutation release exosomes that can transfer the mutant protein to spinal neurons, thereby inducing selective motor neuron death (Basso et al. 2013). Supporting this, astrocyte‐derived exosomes in conditioned media reduced overall protein, while exosomes from healthy astrocytes restore mitochondrial metabolic function impaired by complex I deficiency (Calabria et al. 2019). A proteomic analysis of astrocyte‐derived exosomes identified 189 proteins implicated in cell adhesion and anti‐apoptotic regulation. Key components included RNase 4, IGF‐1, Akt, and regulators of pro‐apoptotic pathways such as Bax and cleaved caspase‐3, along with upregulated Bcl‐2 (Bonafede et al. 2019).

CSF‐derived EVs from ALS patients also show disease‐relevant proteomic alterations. It has been found no significant differences in EV size or concentration, but reduced expression of proteasome core complex proteins, including bleomycin hydrolase, indicating impaired proteostasis (Thompson et al. 2020). Additionally, C9orf72 mutation carriers displayed specific changes such as upregulation of UBA1, although longitudinal analyses revealed no temporal shifts in EV protein levels and only modest differences between genetic subtypes (Thompson et al. 2020). Complementary findings in serum‐derived EVs from early‐stage ALS patients showed differential expression of 45 proteins and several lipid metabolites (e.g., sphingomyelin, phosphatidylcholine, and phosphatidylethanolamine), implicating altered lipid metabolism and inflammation (al Ojaimi et al. 2025). Two interomics modules—comprising lipid‐associated proteins and metabolites—were strongly associated with ALS, suggesting their potential as diagnostic biomarkers.

Proteomic profiling of EVs from ALS‐affected brain tissue has also revealed key disease mechanisms. In motor cortex‐derived EVs, 16 differentially expressed proteins were found in ALS, including STAU1 and DHX30—associated with stress granules—as well as decreased VCAM‐1, potentially reflecting late‐stage disease (Vassileff et al. 2020). In a plasma EV study, the investigators identified seven upregulated proteins (FIBA, FIBB, FIBG, C09, VWF, LBP, and PRG‐4), with PRG‐4 correlating with preserved cognitive function in ALS patients, positioning it as a candidate biomarker for disease monitoring and a possible neuroprotective agent (Vilardo et al. 2024).

Further highlighting the importance of EV subtypes, it was found that while overall EV concentration did not differ between ALS patients and controls, microvesicles in ALS showed increased size and carried pathological proteins (e.g., SOD1, TDP‐43, p‐TDP‐43, FUS), suggesting a role in prion‐like propagation (Sproviero et al. 2018). In contrast, exosomes were enriched in SOD1 but showed no significant protein‐level changes. A related study found that serum and CSF EVs from sporadic ALS patients exhibited elevated inflammation‐related proteins and reduced unfolded protein response proteins, both correlated with disease severity (Kato et al. 2024). These findings led to the use of ropinirole hydrochloride to reverse these signatures, highlighting its anti‐inflammatory effects via astrocytic dopamine D2 receptor signaling (Kato et al. 2024). Furthermore, in this same study, machine learning identified osteoglycin in serum EVs as a promising biomarker of ALS progression.

In a very comprehensive study, 334 proteins were identified with LC–MS/MS in CSF‐derived EVs, including a significant increase in nucleolar complex protein 2 homolog (NIR), which was reduced in ALS motor neurons, suggesting nucleolar stress and impaired apoptosis regulation (Hayashi et al. 2020). Finally, a recent preprint identified nine candidate protein biomarkers in serum EVs from newly diagnosed ALS patients—such as haptoglobin, hemoglobin subunits, complement C8 beta chain, and afamin—implicating dysregulated heme homeostasis, autophagy, and immune response early in the disease (Vassileff et al. 2023). These findings provide a foundation for liquid biopsy‐based diagnostics and support the utility of EV proteomics in understanding ALS pathogenesis and identifying therapeutic targets.

### 5.4 Traumatic Brain Injury (TBI)

Injuries to the CNS, particularly those affecting the brain or spinal cord, often result in irreversible loss of motor and sensory functions, significantly impairing patients' quality of life (Glotfelty et al. 2019). Among such injuries, TBI is a leading cause and is defined as brain trauma resulting from external mechanical forces applied to the head. Unlike the CNS, the peripheral nervous system (PNS) demonstrates robust regenerative capacity, attributed in part to the regenerative functions of Schwann cells (SCs). In contrast, CNS regeneration is hampered by the low plasticity of mature neurons and a hostile post‐injury environment (O'Shea et al. 2024).

Interestingly, SCs have been transplanted into the CNS in animal models to promote regeneration after injury. However, one major limitation is the low post‐transplantation survival rate of SCs, primarily due to apoptosis mediated by p75NTR signaling (Ahmad et al. 2015). A promising alternative to cell transplantation involves harnessing the regenerative capacity of SC‐derived EVs, particularly exosomes. In a recent study, the protein cargo of exosomes derived from primary SC cultures was characterized using LC–MS, with Mascot database searches identifying 433 proteins consistently across three biological replicates (Wei et al. 2019). Notably, 91.92% of these proteins matched entries in the ExoCarta database, confirming the successful enrichment of exosomes. Among them, αB‐crystallin and Galectin‐1 were associated with axonal regeneration and inhibition of inflammation, respectively.

The utility of EVs in TBI extends beyond diagnosis. Their unique ability to cross the BBB and deliver biologically active cargo—including proteins, lipids, and nucleic acids—makes them promising tools for monitoring and modulating disease progression. This is particularly relevant in the context of long‐term neurodegenerative outcomes such as AD and PD. A comprehensive review by (Karnati et al. 2019) emphasizes the diagnostic potential of neuron‐derived EVs isolated from blood, saliva, and urine. These vesicles often contain validated biomarkers such as GFAP, S100B, and UCH‐L1, and may serve as vehicles for therapeutic intervention by transporting RNAs and proteins to target cells.

A study analyzing exosome‐enriched EVs isolated from the CSF of 15 National Football League (NFL) players exhibiting cognitive and neuropsychiatric symptoms, compared to 16 asymptomatic controls, employed SDS‐PAGE followed by tandem mass tag (TMT) 10‐plex labeling and nanoLC–MS/MS analysis. The proteomic analysis identified a total of 429 proteins, among which 62 were abundantly present in EVs from symptomatic NFL players. Functional annotation using the DAVID database revealed that 73.9% of the identified proteins were classified as exosomal, and 11.8% were enriched in pathways associated with AD or aging‐related processes (Muraoka et al. 2019).

It has also been shown that severe TBI leads to a marked increase in the release of EVs into the CSF (Manek et al. 2018). These vesicles were smaller and contained distinct protein profiles compared to those from control individuals. In the proteomic analysis, 466 proteins were identified in TBI‐derived EVs, including markers of cytoskeletal integrity, synaptic function, extracellular matrix remodeling, and cell death (Manek et al. 2018). Known TBI biomarkers such as GFAP, UCH‐L1, synaptophysin, and αII‐spectrin breakdown products were significantly enriched. Systems biology analyses revealed enrichment in pathways involved in oxidative stress, neuronal death, axonal injury, and cytoskeletal dynamics.

Studies examining EVs isolated from the plasma of TBI patients have further demonstrated their potential as biomarkers. In one investigation, patients with altered consciousness (Glasgow Coma Scale ≤ 14) exhibited significantly higher levels of GFAP in plasma‐derived EVs compared to controls and patients with normal consciousness (Puffer et al. 2020). Moreover, 11 differentially expressed microRNAs (miRNAs) were identified, several of which are implicated in pathways regulating cell survival, apoptosis, and nervous system functions. Although the study was limited by small sample size and the inability to isolate brain‐specific EVs, it highlights the promise of circulating EVs for real‐time monitoring of brain injury and recovery.

### 5.5 Stroke

Recent proteomic studies on circulating EVs in stroke patients have revealed critical pathway dysregulations contributing to neuronal damage and recovery. A 2024 case–control study of serum EVs from ischemic stroke patients demonstrated that diabetic stroke (DS) patients exhibited exacerbated activation of the complement cascade compared to non‐diabetic stroke (nDS) individuals (Qadri et al. 2024). The EV cargo in DS was enriched in pro‐thrombotic and inflammatory proteins, such as fibrinogen chains (FIBG/FIBB) and the NF‐κB regulator IKKε, while showing a relative depletion of neuroprotective factors. These findings suggest that diabetes amplifies stroke‐induced EV signals linked to inflammation and coagulation, thereby potentially worsening neuronal injury.

Proteomic comparisons of EVs in different stroke subtypes indicate that their cargo reflects the anatomical and pathological extent of injury. In cortical strokes, EVs were enriched in proteins associated with neurite outgrowth and neurogenesis (e.g., GAP43, NCAM1), suggesting activation of endogenous repair processes. However, these EVs also contained elevated levels of pro‐inflammatory mediators such as C1QA, highlighting their dual role in both injury propagation and inflammation resolution. In contrast, EVs from subcortical strokes were enriched in proteins linked to anti‐inflammatory signaling and vascular stability (e.g., annexins, HSP70), and were associated with reduced blood–brain barrier disruption (Otero‐Ortega et al. 2021).

Beyond identifying biomarkers, EV proteomics has highlighted specific molecules that actively mediate damage or repair. For example, in a preclinical model, EVs derived from neural progenitor cells (NPCs) under ischemic‐like stress were shown to carry axon growth and guidance proteins, such as IQGAP2, Trio, and Rab7, alongside cues like Semaphorin‐6A and Ephrin‐A5, the latter of which is implicated in vascular remodeling post‐stroke (Campero‐Romero et al. 2023). This supports the view of EVs as vectors of regenerative signaling that facilitate cytoskeletal reorganization and axonal repair after ischemic injury. Similarly, astrocyte‐derived EVs have demonstrated therapeutic potential. A single intracerebroventricular injection of astrocyte EVs into rats subjected to focal ischemia enhanced white matter remodeling and improved motor recovery (Heras‐Romero et al. 2022). These effects are consistent with proteomic studies, which show that astrocyte EVs are enriched in pro‐growth and pro‐survival proteins (Bernal Vicente et al. 2025). Furthermore, in a meta‐analysis of hypoxia‐induced astrocyte EV proteomes aligned with proteomic profiles from 16 stroke‐related datasets, we identified overlapping proteins such as APOE, STAT3, HSP90 isoforms, annexins, and 14–3‐3 proteins—implicated in stress responses, synaptic plasticity, and cytoskeletal remodeling (Bernal Vicente et al. 2025).

In addition to neural and glial cells, immune cell–derived EVs also contribute to secondary brain injury. Neutrophil‐derived exosomes, for instance, have been shown to compromise the BBB by downregulating tight junction proteins (Claudin‐5, Occludin, ZO‐1) and transferring microRNAs that deregulate endothelial integrity (Tang et al. 2023).

Clinical EV proteomics has also uncovered distinct signatures associated with different stroke types and outcomes. In patients with intracerebral hemorrhage (ICH), EVs collected at 24 h and 7 day post‐event revealed differential protein expression linked to prognosis. Improved neurological recovery was associated with proteins involved in neurogenesis and stress response (e.g., DERA, VNN2, TOMM34), while limited recovery outcomes correlated with inflammation markers such as CRP and SAA2 (Casado‐Fernández et al. 2024). Similarly, proteomic analyses of mEVs from convalescent plasma of lacunar infarction (LACI) patients identified 573 proteins, many associated with oxygen–glucose deprivation, vesicular trafficking, and iron metabolism. Limited recovery was linked to broader proteome dysregulation, and 63 candidate biomarkers were proposed, expanding the diagnostic landscape for LACI (Datta et al. 2022).

Efforts to identify predictive biomarkers before stroke onset have also leveraged EV proteomics. In a prospective study, serum EVs analyzed by iTRAQ‐based proteomics showed that four proteins—alpha‐2‐macroglobulin (A2MG), complement subcomponents C1QB and C1R, and histidine‐rich glycoprotein (HRG)—were significantly elevated in individuals who later developed ischemic stroke (Mitaki et al. 2021). In parallel, remote ischemic preconditioning (RIPC) has been shown to alter the proteomic and metabolomic signatures of serum exosomes. In a 2023 study, RIPC‐modulated EVs were enriched in metabolites and proteins associated with neuroprotective pathways, including sphingolipid metabolism, serotonergic signaling, and oxidative phosphorylation. Notably, candidates such as ApoA1 and hemopexin came up as potential biomarkers of ischemic tolerance (Du et al. 2023).

Altogether, these findings illustrate the transformative potential of EV proteomics in stroke research. EVs capture dynamic molecular events that govern both damage and recovery—from inflammation, BBB disruption, and apoptosis to neurogenesis, plasticity, and angiogenesis. Many EV proteins, including C1q, semaphorins, heat shock proteins, and annexins, represent novel mechanistic targets with diagnostic and therapeutic promise. EVs are not merely biomarkers; they are active participants in stroke pathology and repair, offering unique opportunities to understand and modulate the molecular landscape of brain injury.

## 6 Discussion

EVs' proteomic and molecular profiling are accelerating the discovery of biomarkers across neurological diseases. From AD to PD to ALS, disease‐associated EV cargo (proteins, phosphorylated epitopes, etc.) reflects the molecular hallmarks of pathology. Ongoing advances in EV isolation (e.g., improved CSF EV yields Kangas et al. 2023) and sensitive cargo detection (such as single‐vesicle analysis methods) are expected to refine these biomarker signatures further. Ultimately, panels of EV‐derived markers may enable earlier and more accurate diagnosis, for instance, by detecting misfolded protein aggregates or neuroinflammatory signals in patients' blood years before overt symptoms appear.

Beyond biomarkers, EVs are providing mechanistic insights into how neurological diseases initiate and spread. A consistent theme in recent research is the role of EVs in propagating misfolded, pathogenic proteins throughout the nervous system. In AD and related dementias, EVs appear to facilitate the cell‐to‐cell spread of tau and amyloid proteins in a “prion‐like” manner (Sattarov et al. 2024). For example, cryo‐electron microscopy analyses published in 2025 have visualized filamentous tau enclosed within EVs from AD brain tissue (Fowler et al. 2025). These tau filaments—composed mainly of truncated, aggregation‐prone tau—were tethered to the inner membrane of EVs, suggesting selective packaging of pathological tau into vesicles (Fowler et al. 2025). The association of assembled tau with endosomal‐origin EVs supports the idea that neurons may expel tau aggregates via exosomes, which other cells can take up (Fowler et al. 2025). This EV‐mediated tau secretion could be a double‐edged sword: it may aid in the clearance of toxic tau and seed tau pathology in neighboring cells. Indeed, experimental studies have shown that EVs carrying pathological tau can induce tau misfolding and accumulation in recipient cells and animal models (Hook et al. 2023). Such evidence positions EVs as active vectors in tauopathies, such as AD and CTE, the head injury‐associated tauopathy, helping to explain the stereotyped spread of tau neurofibrillary tangles through connected brain regions.

A similar propagation role is seen in synucleinopathies (PD and related disorders). α‐Synuclein aggregates can be packaged into EVs and transported across cell boundaries, effectively disseminating Lewy body pathology. A 2024 study of over 140 PD patients' blood found that although the total number of circulating EVs was reduced in PD, there was a marked increase in the filamentous α‐synuclein contained within them (Ishiguro et al. 2024). These α‐synuclein fibrils in EVs were elevated in PD compared to healthy controls and other Parkinsonian conditions like multiple system atrophy (Ishiguro et al. 2024). Notably, the study provided direct evidence that EVs can transfer misfolded α‐syn from the peripheral to the central nervous system: EV‐associated α‐syn seeds were shown to travel from the bloodstream to the brain, where neurons can take them up. This supports a model in which peripheral tissues might contribute to brain pathology via EV trafficking of α‐syn, offering a new mechanistic link between systemic pathology and PD's central neurodegeneration. Overall, these findings stress EVs as critical “cargo ships” for pathogenic proteins, helping to explain the progressive, network‐based spread of lesions in PD.

In ALS and FTD, EVs are also implicated in the spread of toxic proteins, such as TDP‐43 or SOD1, between cells. Neurons and glia releasing EVs loaded with misfolded TDP‐43 might seed pathology in neighboring cells, and indeed, EV‐associated TDP‐43 from ALS patients' brains can induce TDP‐43 aggregation and mislocalization in cultured recipient cells. The consistent presence of misfolded TDP‐43 and other ALS proteins in patient‐derived EVs (Chatterjee et al. 2024), and their correlation with datasets, suggests that EVs participate in the contiguous spread of ALS pathology along the neuroaxis. Notably, in vivo models have demonstrated that reducing EV release, for example, by inhibiting neutral sphingomyelinase, can mitigate the spread of pathological proteins and neurodegeneration.

EVs also contribute to neuroinflammatory mechanisms that exacerbate neurological diseases. Injury and stress conditions, like TBI or neurodegeneration, can provoke cells to release EVs containing inflammatory mediators or nucleic acids that activate immune pathways. One insight is that damaged cells release EVs enriched in cytosolic DNA after TBI, which can bind and activate the cGAS–STING pathway in immune cells (Liu et al. 2023). This DNA‐sensing pathway triggers downstream interferon and NF‐κB signaling, driving chronic neuroinflammation. Thus, TBI‐induced EVs may bridge acute injury and sustained inflammation, propagating immune activation throughout the CNS. Conversely, EVs might also act in a protective capacity: recent evidence indicates that microglia can expel DNA and inflammasome factors inside EVs to prevent their cytosolic buildup (Arvanitaki et al. 2024). Age‐related decline in this EV‐mediated clearance could explain the accumulation of pro‐inflammatory DNA species in the brain with aging. In sum, EVs modulate neuroinflammatory cascades by transporting inflammatory signals, or their regulators, between cells. This dual role—sometimes exacerbating pathology by spreading danger signals, other times mitigating it by sequestering toxic molecules—makes EVs a fascinating target for understanding the neuroimmune nexus in conditions from TBI and CTE to AD.

## 7 EVs as Diagnostic Tools and Therapeutic Targets

The cell‐type specificity and stability of EV cargo make EVs especially attractive for clinical applications in diagnosis and disease monitoring. EV‐based diagnostics are advancing rapidly, with several proof‐of‐concept studies in the past few years. In AD, where definitive diagnosis traditionally requires CSF biomarkers or PET imaging, blood‐derived EV assays offer a less invasive alternative. Researchers have shown that neuronal EVs isolated from blood plasma carry AD‐associated proteins (like P‐tau and Aβ) that mirror brain changes (Sattarov et al. 2024). Plasma neural EV levels of P‐tau_181, Aβ42, and even lysosomal enzymes have been found to differentiate AD patients from controls, reflecting brain pathology in accessible fluids. The aforementioned pilot study of CSF EV P‐tau217/181 is another example, suggesting that refining which EVs, CSF vs. plasma, neuron‐derived vs. glia‐derived, are analyzed can enhance the diagnostic signal (Sattarov et al. 2024). These approaches point toward an EV‐based AD diagnostic that could complement or even replace some CSF tests in the future, while also perhaps indicating the molecular subtype of pathology (e.g., tau versus Aβ load).

In PD, EV assays are pointing toward an earlier and more accurate diagnosis. Since PD pathology often begins years before motor symptoms appear, there is intense interest in identifying individuals at risk. Measuring α‐synuclein in L1CAM‐positive neuronal EVs from blood has shown promise for identifying prodromal PD (Wood 2024). Additionally, EV biomarkers can help distinguish PD from clinically similar disorders. For example, as noted above, astrocyte‐derived EV α‐synuclein was elevated in PD but not in multiple system atrophy, enabling a differential diagnosis between these synucleinopathies (Wang et al. 2023). Such specificity is invaluable, as it could guide patient selection for disease‐specific therapies, for instance, ensuring a patient has PD and not another Parkinsonian syndrome before prescribing an α‐synuclein‐targeting treatment. These studies are increasingly published in high‐impact neurology and translational journals, underscoring the enthusiasm for EV‐based diagnostics in PD. However, as a cautionary note, we must say that although L1CAM has frequently been used as a neuronal marker to isolate EVs from plasma, systematic validation studies have shown that L1CAM is predominantly present in its soluble form rather than being enriched on EV membranes, both in plasma and CSF. Moreover, L1CAM is expressed in non‐neuronal tissues, including gastrointestinal epithelial cells, which further complicates its specificity as a neuronal marker in peripheral biofluids. These findings suggest that attributing plasma EVs to a neuronal origin solely based on L1CAM may lead to overinterpretation (Kadam et al. 2025). Future studies should therefore combine L1CAM with complementary markers, such as light neurofilament, synaptophysin, or other neuron‐specific proteins or RNAs, to more accurately determine the cellular origin of EVs in Parkinson's disease and related conditions.

EVs offer hope even for conditions like CTE, which currently lack an antemortem diagnostic test. CTE is a tauopathy resulting from repetitive head trauma, often in athletes, and at present, it can only be confirmed at autopsy. However, researchers have observed that exosomal tau in plasma might be a proxy for the tau neuropathology in CTE (Gerges et al. 2023). In preliminary studies, elevated levels of phosphorylated tau in plasma EVs correlated with a history of repetitive concussions and with cognitive symptoms in living subjects, suggesting EV tau as a candidate biomarker for CTE. While such findings are early, they demonstrate the broader principle that EVs can carry disease‐defining proteins from the brain into the bloodstream. With sensitive assays, these EV cargoes can be measured to infer the presence of pathologies like diffuse tau tangles in CTE, potentially enabling diagnosis or risk assessment in life. Ongoing longitudinal studies in contact‐sport athletes examine whether an “EV biomarker signature” (combining tau, inflammatory markers, etc.) can predict CTE years before symptoms, which would be a transformative clinical tool.

In acute injuries like TBI, EVs in blood may aid in grading injury severity and predicting outcomes. Because EVs can cross the BBB, they can ferry brain injury markers into the circulation. Recent research suggests that measuring known TBI protein biomarkers (such as GFAP or neurofilament) specifically within circulating EVs improves the sensitivity for detecting mild TBI compared to measuring them in whole plasma (Dong et al. 2023). Brain‐derived EVs isolated by neuron‐ or glia‐specific markers contain a concentrated repository of injury signals and may distinguish TBI‐induced changes from peripheral confounders. Indeed, a 2023 review noted that “brain‐derived EVs support more specific screening” for TBI diagnosis and prognosis than bulk biofluid analysis (Dey et al. 2023). This has given rise to the EV‐based “theragnostic” signature in TBI, combining diagnostic biomarkers with ongoing damage or repair indicators that could inform therapy selection. Such approaches might enable, for example, rapid blood tests in concussion clinics to detect subtle neurotrauma or to monitor recovery and risk of neurodegenerative sequelae like CTE after repetitive injuries.

## 8 Therapeutic Applications of EVs in Neurology

EV research is also laying the groundwork for novel therapeutic strategies. EVs are being explored as therapeutic agents and drug delivery vehicles. One major avenue is using stem cell–derived EVs as a cell‐free therapy for neurodegenerative diseases or brain injury. Investigators reason that EVs can convey many of the beneficial paracrine signals of stem cells without the risks of cell transplantation. Exciting results were reported in late 2024 using EVs derived from human induced pluripotent stem cell–derived neural stem cells (hiPSC‐NSCs) to treat an AD preclinical model (Madhu et al. 2024). In this study, EVs enriched with neuroprotective and immunomodulatory cargo were administered intranasally (a delivery route that targets the CNS) in 5xFAD transgenic mice. The EV treatment produced a remarkable reduction in pathology, dampening proinflammatory gene programs in microglia (including the NF‐κB and NLRP3 inflammasome pathways), reducing reactive astrocyte changes, and leading to lower amyloid plaque burden and phosphorylated tau levels in the brain. Mice treated with the AD model showed improved cognitive and memory performance compared to controls. These results demonstrate that EVs can recapitulate a therapeutic effect in vivo against AD pathology by reprogramming the brain's inflammatory and protein aggregation milieu (Madhu et al. 2024). They also highlight practical advantages of EVs—being non‐immunogenic and capable of widespread diffusion—in reaching diseased brain cells that many drugs cannot.

Similar therapeutic potential is being explored in other conditions. In models of stroke, administration of astrocyte–derived EVs has been shown to improve functional recovery in part by delivering microRNAs and proteins that foster neurorepair (Heras‐Romero et al. 2022). EVs can be engineered to carry specific therapeutic cargo, such as loading EVs with neurotrophic factors, anti‐apoptotic proteins, or RNA interference molecules targeting mutant genes. Because EVs are biocompatible and can cross the BBB, they are natural nanoscale delivery systems. Early‐phase studies have even functionalized EV surfaces with targeting ligands (e.g., RVG peptide to target neurons) to enhance delivery specificity to the brain (Dey et al. 2023). While these approaches remain preclinical, EV‐based drug delivery is gaining traction for disorders such as PD (e.g., delivering GDNF or siRNAs to dopaminergic neurons) and glioblastoma (administering chemotherapy or gene therapy directly into invasive tumor cells). Furthermore, the ability to inhibit or modulate EV production is being examined as a therapeutic strategy—for example, could reducing EV release slow the spread of pathology in synucleinopathies or tauopathies? Some studies have utilized pharmacological inhibitors of EV biogenesis to successfully mitigate the propagation of toxic protein species in cell models, suggesting a potential disease‐modifying strategy for the future.

Therapeutically, EVs derived from human umbilical cord mesenchymal stem cells (hUC‐MSC‐EVs) have shown beneficial effects in APP/PS1 transgenic mice. Treated animals exhibited improvements in memory, learning, and motor performance, with proteomic normalization of AP2A1 and AP2B1—key proteins in the synaptic vesicle cycle. These findings suggest that hUC‐MSC‐EVs modulate synaptic function and represent a viable intervention strategy (Li et al. 2024).

EVs themselves might be targets of therapy—for instance, immunotherapy approaches are being conceived where antibodies or receptors could bind and neutralize pathogenic EVs in circulation (Kuang et al. 2025). An illustrative example outside neurology showed that antibody‐coated EVs can be directed to specific cell types (Wiklander et al. 2024), raising the possibility of similarly tagging EVs that carry neurotoxic cargo for clearance. Though speculative, one could imagine boosting the brain's EV‐mediated clearance mechanisms (such as enhancing microglial EV expulsion of protein aggregates or DNA) to alleviate neurodegeneration. All these strategies, from using EVs as therapeutics to targeting EV pathways, stem from recognizing that EVs are central players in the brain's intercellular communication network. Harnessing or modulating this network offers a fresh therapeutic paradigm.

The engineering of EVs to deliver siRNAs, neurotrophic factors, or other therapeutic cargos, as well as the challenges related to manufacturing scalability and regulatory approval, are key topics that have been extensively and very recently reviewed elsewhere, including dedicated overviews of engineered exosomes for CNS disorders and drug delivery across the BBB (Lee et al. 2025), focused analyses of strategies to optimize EV cargo loading and therapeutic delivery (René and Parks 2024), and comprehensive discussions of clinical translation, good manufacturing practices (GMP)‐compliant, and regulatory frameworks for EV‐based therapeutics (Xu et al. 2025). Moreover, preclinical work demonstrates that engineered EVs can exert therapeutic effects in neurological disorders, particularly through strategies that enhance CNS targeting and improve the loading and delivery of therapeutic cargo (Cheng et al. 2023). Advanced EV‐mediated delivery systems tailored for neurological conditions now incorporate strategies to enhance vesicle stability, extend systemic circulation, and increase effective penetration into the brain (Ahmed et al. 2023). Finally, (Yao et al. 2024) provide an in‐depth overview of recent advances in therapeutic EV engineering, including innovations in surface modification, enhanced cargo‐loading strategies, and next‐generation design approaches aimed at increasing therapeutic efficacy. Taken together, these reviews compile a substantial body of evidence demonstrating that EV‐based therapeutics are advancing rapidly toward increasingly sophisticated, highly targeted, and multifunctional platforms.

Despite this progress, significant translational hurdles remain. Manufacturing scalability, batch‐to‐batch variability, EV heterogeneity, and the need for GMP‐compliant production pipelines represent major challenges for clinical‐grade EV preparation. Regulatory considerations are likewise complex, particularly given the hybrid nature of engineered EVs—partially biological and partially manufactured. Characterizing purity, stability, potency, and mechanism of action becomes even more critical as EVs progress toward clinical trials. In addition, defining standardized release criteria and ensuring reproducibility across manufacturing sites remain unresolved obstacles. These limitations make evident the need for rigorous methodological standardization, harmonized analytical workflows, and transparent reporting—elements that closely align with ISEV recommendations and are essential for advancing EV‐based therapies from preclinical promise to clinical reality in neurological disease.

In this context, it becomes evident that proteomic insights will play an increasingly important role in guiding the rational design of next‐generation therapeutic EVs. Identifying protective, pro‐regenerative, or disease‐modifying proteins within EV cargo helps inform engineering strategies and clarify the mechanisms underlying EV‐mediated therapeutic effects. Integrating proteomics with therapeutic EV development, therefore, represents an important future direction for the field.

## 9 Conclusion

EVs have moved to the forefront of neuroscience research as both mechanistic instigators of neurological disease and promising tools for clinical innovation. Recent studies (2023 onwards) provide compelling evidence that EVs carry the molecular signatures of neurodegenerative and neurotraumatic conditions—from misfolded proteins like tau, α‐synuclein, and TDP‐43 to inflammatory signals—and actively participate in disease processes by spreading pathology and modulating cell responses. These properties make EVs rich reservoirs of biomarkers, enabling advances in early diagnosis and disease stratification in AD, PD, ALS, and beyond. Equally exciting is the translational leap of EVs into therapeutics: leveraging their natural delivery capabilities to either remove harmful signals or deliver beneficial ones to the brain. While challenges remain, such as standardizing EV isolation and ensuring cargo specificity, the progress of the past few years has solidified EV research as a bridge between mechanistic understanding and clinical application in neurology. Continued interdisciplinary efforts—integrating proteomics, nanotechnology, and clinical trials—will likely further unravel the roles of EVs in neurological pathologies and unlock new diagnostic and therapeutic strategies, bringing us closer to precision medicine for complex brain disorders.

## Author Contributions

**Berenice N. Bernal‐Vicente:** investigation, data curation, writing – original draft, conceptualization. **Isaac Ponce:** investigation, writing – original draft, data curation. **Emmanuel Ríos‐Castro:** investigation, writing – original draft, methodology, writing – review and editing, formal analysis. **Perla Moreno‐Castilla:** investigation, visualization, validation. **Luis B. Tovar‐y‐Romo:** conceptualization, investigation, funding acquisition, writing – original draft, writing – review and editing, supervision, data curation, validation, formal analysis, project administration, resources.

## Funding

This work was supported by Dirección General de Asuntos del Personal Académico, Universidad Nacional Autónoma de México, IN214723.

## Data Availability

This review article does not include new datasets generated or analyzed by the authors. All data discussed in this review work are derived from previously published studies, which are cited throughout the manuscript.
